# Effect of Biodiversity Changes in Disease Risk: Exploring Disease Emergence in a Plant-Virus System

**DOI:** 10.1371/journal.ppat.1002796

**Published:** 2012-07-05

**Authors:** Israel Pagán, Pablo González-Jara, Alejandra Moreno-Letelier, Manuel Rodelo-Urrego, Aurora Fraile, Daniel Piñero, Fernando García-Arenal

**Affiliations:** 1 Centro de Biotecnología y Genómica de Plantas (UPM-INIA), and E.T.S.I. Agrónomos, Campus de Montegancedo, Universidad Politécnica de Madrid, Pozuelo de Alarcón, Madrid, Spain; 2 Departamento de Ecología Evolutiva, Instituto de Ecología, Universidad Nacional Autónoma de México, Ciudad de México, México; University of California Riverside, United States of America

## Abstract

The effect of biodiversity on the ability of parasites to infect their host and cause disease (i.e. disease risk) is a major question in pathology, which is central to understand the emergence of infectious diseases, and to develop strategies for their management. Two hypotheses, which can be considered as extremes of a continuum, relate biodiversity to disease risk: One states that biodiversity is positively correlated with disease risk (Amplification Effect), and the second predicts a negative correlation between biodiversity and disease risk (Dilution Effect). Which of them applies better to different host-parasite systems is still a source of debate, due to limited experimental or empirical data. This is especially the case for viral diseases of plants. To address this subject, we have monitored for three years the prevalence of several viruses, and virus-associated symptoms, in populations of wild pepper (chiltepin) under different levels of human management. For each population, we also measured the habitat species diversity, host plant genetic diversity and host plant density. Results indicate that disease and infection risk increased with the level of human management, which was associated with decreased species diversity and host genetic diversity, and with increased host plant density. Importantly, species diversity of the habitat was the primary predictor of disease risk for wild chiltepin populations. This changed in managed populations where host genetic diversity was the primary predictor. Host density was generally a poorer predictor of disease and infection risk. These results support the dilution effect hypothesis, and underline the relevance of different ecological factors in determining disease/infection risk in host plant populations under different levels of anthropic influence. These results are relevant for managing plant diseases and for establishing conservation policies for endangered plant species.

## Author Summary

Biodiversity has been proposed as a major ecological factor determining disease prevalence. However, the relationship between biodiversity and disease risk remains underexplored. Few studies focus on host-virus systems and, particularly on plant viruses. To address this subject the prevalence of virus infection and disease symptoms was monitored in wild-pepper (chiltepin) populations under different levels of human management. For these populations, species diversity, host genetic diversity and host plant density were determined. Higher levels of human management resulted in increased disease and virus infection risk, which was associated with decreased habitat species diversity and host genetic diversity, and with increased host plant density. More specifically, for wild chiltepin populations, species diversity of the habitat was the primary predictor of disease risk; and host genetic diversity was the primary predictor in managed populations, with host density being generally a poorer predictor of disease risk. These results support a dilution effect of biodiversity on disease risk, and underline the relevance of different ecological factors in determining disease risk in wild and in human-managed habitats.

## Introduction

Understanding the relationship between the risk of infectious diseases and host ecology is a long-standing goal of biological research, central for the management of current infectious diseases and for preventing the emergence of new ones. Indeed, changes in host ecology are among the most frequently identified causes of disease emergence (i.e. the increase of disease incidence following its appearance in a new, or previously existing, host population) [1]–[3]. Because infectious diseases involve interactions between at least two species, it has been proposed for a long time that ecosystem biodiversity will play a key role in disease risk. Current declines in biodiversity have been proposed to be linked with the emergence of infectious diseases, which have fueled a renewed interest on this subject [4]. Two major hypotheses with different predictions relate biodiversity to disease risk. The “Amplification Effect” hypothesis predicts that diversity will be positively correlated with disease risk, as it will result in increased abundance of inoculum sources for a focal host. The “Dilution Effect” hypothesis predicts a negative correlation between biodiversity and disease risk, as a reduction in diversity could result in an increased abundance of the focal host species facilitating disease transmission [5]. These two hypotheses can be considered to represent extremes of a continuum, as the effects of diversity on disease risk would be related to the host range of the pathogen: an Amplification Effect would require a generalist pathogen, while the more restricted the host range of the pathogen, or the higher the differences between shared hosts in their ability to amplify or transmit the pathogen, the higher the Dilution Effect. Increasing evidence derived from pathogens with broadly different life-styles indicates that biodiversity reductions most often result in increased disease risk [4].

The idea linking biodiversity with disease risk is not new in animal or plant pathology. Two classical hypotheses in plant pathology state that the high impact of plant diseases in crops is associated with: i) the reduced species diversity, and higher host density, of agroecosystems as compared to wild ecosystems [6]; ii) the reduced genetic diversity of crops as compared to their wild ancestors or relatives [7]. However, despite that a number of recent studies on the ecology of plant diseases have been added to those dating from the 1980s, support for these hypotheses is still often circumstantial [8]. Attention has focused on analyses of foliar diseases caused by fungi, which mostly indicate that increased biodiversity reduces disease risk [9]–[14]. Remarkably, there are fewer reports referring to viral diseases, which represent a large fraction of emergent plant pathogens [15], and may differ from fungal ones in their relationship to biodiversity. While most plant pathogenic fungi are directly transmitted specialists [16], most plant-infecting viruses are vector transmitted, and are host generalists but often vector specialists [17]. Most studies with plant viral diseases have focused on generalist viruses infecting grasses, generally finding an amplification effect [18]–[21]. Interestingly, work on plant diseases largely failed to assess the role of various possible mechanisms by which reduced biodiversity may affect disease risk (but see [10], [11], [22]). Particularly, it is often difficult to differentiate the effects of increased host density and of reduced species diversity [4]. Hence, there is a need of research aimed at analyzing the effects of biodiversity on plant disease risk and, specifically, at disentangling the role of the various factors associated to ecosystem diversity. This is the goal of the present work.

The focal host in this study is the wild pepper *Capsicum annuum* var. *glabriusculum* (Dunal) Heiser and Pickersgill [23], also known as “chiltepin”. Chiltepin is found in Mexico in a variety of habitats from the Yucatan peninsula and the Gulf of Mexico to the Sonoran desert [24], [25]. Chiltepin is a deciduous, perennial bush that grows for 5–8 years and vegetates and reproduces during the rainy season. Birds disperse the seeds from its red pungent fruits [24]. Human harvesting of fruits from wild chiltepin plants is a common practice in central and northern Mexico [26], [27]. A second level of human exploitation involves tolerance or favoring the growth of spontaneously dispersed chiltepin plants in anthropic habitats, such as pastures and living fences (i.e., let-standing plants, *sensu* [28]). Last, chiltepin cultivation in home gardens or in small traditional plots has started in the recent past [25]. Cultivation has not yet lead to domestication, and cultivated chiltepin populations, which are managed as annual crops, do not show obvious phenotypic differences with wild ones [25]. Wild chiltepin populations show a large genetic variation and a strong spatial structure associated with the biogeographical province of origin, and human management results in a significant loss of both spatial structure and genetic diversity [25]. This habitat diversity makes chiltepin a uniquely good system to analyze the relationship between biodiversity and disease risk.

We focused on two contrasting pepper-infecting virus groups. The first involves two species of the genus *Begomovirus* (*Geminiviridae*): *Pepper golden mosaic virus* (PepGMV), and *Pepper huasteco yellow vein virus* (PHYVV), here treated collectively as “begomoviruses”. These species have a two-segmented single-stranded (ss) DNA genome; narrow host ranges limited in nature mostly to species of the genera *Capsicum*, *Solanum* and *Datura* (*Solanaceae*), and are transmitted in a persistent manner by the whitefly *Bemisia tabaci* Gennadius (Homoptera, Aleyrodidae) [29]–[31]. The B biotype of *B. tabaci*, characterized by a broad plant host range, a high reproductive potential, and a high efficiency as a vector for begomoviruses, is prevalent in Central and North America [32]. The second virus is *Cucumber mosaic virus* (CMV), genus *Cucumovirus* (*Bromoviridae*), with a tripartite, ssRNA genome, and a typical generalist, infecting more than 1000 species of both mono- and dicotyledonous plant families. CMV is transmitted in a non-persistent manner by more than 80 species of aphids, thus being also a vector generalist [33].

Utilizing these host-pathogen systems we specifically addressed if: i) modification of chiltepin habitat associated with different levels of human management resulted in changes in disease or infection risk, ii) reduction of species diversity increases disease or infection risk, iii) decreased host genetic diversity had an effect on disease or infection risk, iv) increased host plant density resulted in increased disease or infection risk and v) the above effects were different for viruses with different life-histories.

To answer these questions, we visited over three years neighboring wild, and human managed (i.e., of let standing and cultivated plants) chiltepin populations from different biogeographic provinces in Mexico. For each population, species diversity, host density and the prevalence of plants showing symptoms of virus infection were quantified in the field as an estimate of disease risk. Plants were collected at each population and their status (infected/non-infected by several viruses) was determined in the laboratory in order to estimate infection risk. Results indicate that disease and begomovirus infection risks, but not CMV infection risk, decrease with increasing biodiversity. We propose that observed differences between begomovirus and CMV infection risk can be due to different transmission modes.

## Materials and Methods

### Field Surveys and Plant Collections

**Figure 1:**
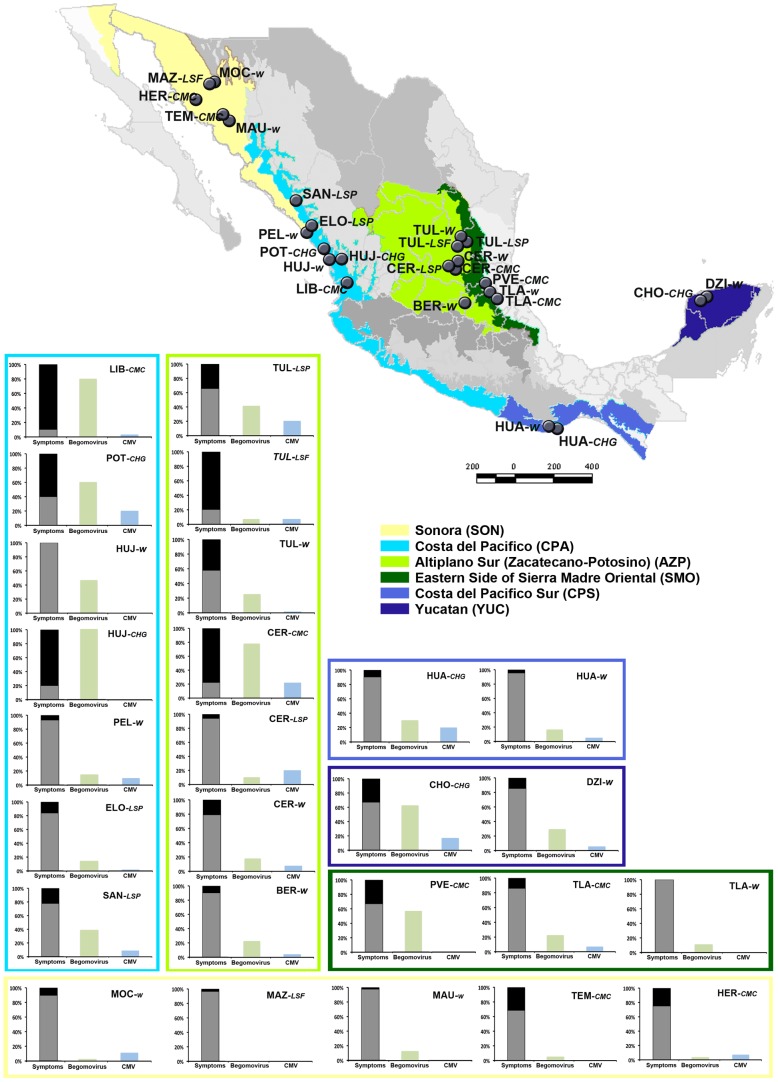
Geographic location of chiltepin populations, and prevalence of symptomatic plants, begomoviruses and CMV. Map shows the location of populations from wild (W), let standing (LSP, LSF), and cultivated (CMC, CHG) populations within six biogeographical provinces in Mexico. Bar graphics show the average prevalence of symptomatic (grey) and asymptomatic (black) plants, as well as the prevalence of begomovirus (green) and CMV (blue) infection, for each chiltepin population. Boxes group populations from the same biogeographical province, and are colored accordingly.

Chiltepin populations were visited during the summers of 2007 to 2009 at different sites over the species distribution range in Mexico (Figure 1 and Table 1). A total of 26 populations were localized in different habitats representing three levels of human management: i) ten wild populations (W) in which fruit gathering by local people may occur; ii) six populations of let-standing plants (here from called “let-standing populations”), in anthropic habitats, either pastures (LSP) or live fences (LSF), in which chiltepin plants are tolerated or favored, and iii) ten cultivated populations (C) either at home gardens (CHG) or at small monocultures (CMC). Population sites were assigned to 6 biogeographical provinces: Yucatan (YUC), Eastern side of the Sierra Madre Oriental (SMO), Altiplano Zacatecano-Potosino (AZP), Costa del Pacífico (CPA), Costa del Pacífico Sur (CPS) and Sonora (SON) [34]. A total of 14 populations, located in YUC, SMO, AZP, CPA and CPS, were visited during 2007 and 2008. The 2009 survey was extended to other populations of these five biogeographical provinces and SON, to a total of 26 populations (Table 1). Populations were visited between the 15 of July and the 30 of August, in an attempt to homogenize plant phenology among locations at the stage of flowering and beginning of fruit setting. Due to the highly unpredictable rain regime at some regions, or to extinction, not all populations could be surveyed for the three years.

**Table 1:** Prevalence of virus-like symptoms (i.e., mosaic, leaf curl, leaf lamina reduction, and/or stunting) in the analyzed Mexican chiltepin populations monitored in July–August of 2007 to 2009.

Habitat^1^	Location^2^	Code^3^	Region^4^	2007				2008				2009			
				N^5^	As^6^	S^7^	% sint^8^	n	As	S	% sint	n	AS	S	% sint
Wild	Dzibilchaltun (YUC)	DZI-W	YUC	39	35	4	10.3	57	47	10	17.5				
Wild	Huatulco (OAX)	HUA-W	CPS	36	35	1	2.8	26	24	2	7.7	26	25	1	3.9
Wild	Tlacuapa (SLP)	TLA-W	SMO	9	9	0	0								
Wild	Tula (TAM)	TUL-W	AZP					30	3	27	90	46	41	5	10.8
Wild	Bernal (QRO)	BER-W	AZP	86	66	20	23.3	113	101	12	10.6	156	153	3	1.9
Wild	Cerritos (SLP)	CER-W	AZP	5	4	1	20					14	11	3	21.4
Wild	El Huajote (SIN)	HUJ-W	CPA									13	13	0	0
Wild	Puente Elota (SIN)	PEL-W	CPA	50	42	8	16	45	44	1	2.2	34	34	0	0
Wild	Moctezuma (SON)	MOC-W	SON									79	78	1	1.3
Wild	Los Mautos (SON)	MAU-W	SON									81	79	2	2.5
Let standing	Tula (TAM)	TUL-LSF	AZP									44	9	35	79.5
Let standing	Tula (TAM)	TUL-LSP	AZP					13	5	8	61.5	22	18	4	18.2
Let standing	Cerritos (SLP)	CER-LSP	AZP									33	31	2	6.1
Let standing	Elota (SIN)	ELO-LSP	CPA	54	38	16	29.6	39	36	3	7.7	43	40	3	7
Let standing	Sanalona (SIN)	SAN-LSP	CPA	24	16	8	33.3					21	19	2	9.5
Let standing	Mazocaui (SON)	MAZ-LSF	SON									29	28	1	3.5
Cultivated	Cholul (YUC)	CHO-CHG	YUC	18	10	8	44.4	47	35	12	25.5	11	6	5	45.5
Cultivated	Huatulco (OAX)	HUA-CHG	CPS	10	8	2	20	101	93	8	7.9	24	21	3	12.5
Cultivated	Tlacuapa (SLP)	TLA-CMC	SMO	9	7	2	22.2	46	41	5	10.9	45	38	7	15.6
Cultivated	PuertoVerde (SLP)	PVE-CMC	SMO	17	10	7	41.2	20	3	17	85	81	66	15	18.5
Cultivated	Cerritos (SLP)	CER-CMC	AZP									9	2	7	77.8
Cultivated	El Potrero (SIN)	POT-CHG	CPA									10	4	6	60
Cultivated	El Huajote (SIN)	HUJ-CHG	CPA									10	2	8	80
Cultivated	La Libertad (NAY)	LIB-CMC	CPA									29	3	26	89.7
Cultivated	Temporal (SON)	TEM-CMC	SON									38	26	12	31.6
Cultivated	Hermosillo (SON)	HER-CMC	SON									28	21	7	25
**TOTAL**				**357**	**280**	**77**	**21.6**	**537**	**432**	**105**	**19.6**	**926**	**776**	**150**	**16.2**

1 Habitats belonged to three levels of human management.

2 State is indicated in parenthesis: NAY = Nayarit; OAX = Oaxaca; SIN = Sinaloa, SLP = San Luis Potosí, SON = Sonora, TAM = Tamaulipas, YUC = Yucatan.

3 Populations are designated with the first three letters of the name of the nearest village, plus a code indicating the habitat: W = wild, LSP = Let standing, pasture; LSF = Let standing, living fence; CHG = Cultivated, home garden; CMC = Cultivated, monoculture.

4 Region designates the following biogeographical provinces: YUC: Yucatan; CPS: Costa del Pacífico Sur; SMO: Sierra Madre Oriental; AZP: Altiplano Zacatecano-Potosino; CPA: Costa del Pacífico; SON: Sonora.

5 Total number of plants in the population, except for. BER-w, PEL-w, MOC-w and MAU-w, in which N indicates number of plants sampled (see Material & Methods).

6 Number of asymptomatic plants.

7 Number of symptomatic plants.

8 Percentage of symptomatic plants.

At each location, the following information was collected: 1) The census of the chiltepin population. 2) The status of each censused plant: asymptomatic or showing symptoms commonly related to virus infection (i.e., mosaic, leaf curl, leaf lamina reduction, and/or stunting). 3) The area (m^2^) occupied by the chiltepin population. 4) The inventory of the non-herbaceous vegetation, determined as the number of individuals of each bushy or arboreal species, in the same area of the chiltepin population, to estimate species richness, and evenness according to the Shannon index [35]. Populations BER-w, PEL-w, MOC-w and MAU-w (Table 1) were too large – i.e., more than 200 plants – to census all plants, and both chiltepin censuses and biodiversity inventories were limited to a fixed transect. In this case, the area occupied by the chiltepin population was calculated by prospecting a width of 4 m along the fixed itinerary.

At each population and visit, plants were systematically sampled for laboratory analyses. Plants were sampled regardless of their showing or not symptoms: One plant out of every *x* plants was sampled along fixed itineraries, with itinerary length and *x* (0<*x*≤4) depending on population size, 1–3 young branches with fresh leaves were collected per plant.

### Virus Detection and Plant Genotyping

Infection by CMV and by *Potyvirus* species was analyzed by DAS-ELISA, using commercial antisera against CMV or a monoclonal antibody against a highly conserved motif in the coat protein of potyviruses (Agdia Biofords), according to the manufacturer's instructions. Infection by *Chiltepin yellow mottle virus* (ChYMV, *Tymoviridae*) was analyzed by molecular hybridization using a ^32^P-labeled RNA probe complementary to nucleotides 5365–5777 of ChYMV genomic RNA (Accession No. FN563124) [36]. Infection by species of the genus *Begomovirus* was detected by PCR using degenerate primers designed on the alignment of DNA-A sequences of 43 begomovirus species from the New World: BAOPsp (5′-GCGCCCTGCAGGGGCCYATGTAYAGGAAGCC-3′) and BAONsp (5′-GCGCGCGGCCGCGANGCATGNGTACATGCCAT-3′), which amplify a region in the coat protein gene located between nucleotide positions 392 and 884 in the genome of PepGMV (Accession No. AY928512).

Molecular hybridizations and PCR were performed on total nucleic acid preparations from chiltepin leaves extracted by grinding 200 mg of fresh leaf tissues in three volumes of 200 mM Tris-HCl pH 9, 25 mM EDTA, 1% SDS, 400 mM LiCl, followed by phenol-chloroform extraction [25]. Plants genotyped using the set of 9 nuclear microsatellites markers described in [25] were used to estimate genetic diversity of the 26 chiltepin populations.

### Statistical Analyses

Generalized linear mixed models (GLMM) were used to analyze the difference in the prevalence of virus infection (Begomovirus and CMV), and in the frequency of symptomatic plants, according to chiltepin population, biogeographical province and level of human management of the population, considering these factors as fixed effects. The rationale for considering population as a fixed effect is that all the chiltepin populations that we were able to find were included in the analyses, rather than using a random representation of them. The symptom and virus prevalence values determined for each population in the different years were considered as dependent measures; thus, they were treated as repeated measures in the GLMM. This seems the correct approach for wild and let-standing populations, in which at least a subset of the plants sampled over the years were the same, since chiltepin plants live for several years. This might not be so for cultivated populations, in which plants are managed as an annual crop and may change plots over the years. However, we considered that plots from different years were close enough to be spatially correlated, and therefore repeated measures are warranted. In addition, results did not differ when data from cultivated populations were analyzed as independent measures (not shown). To determine whether values of analyzed traits were significantly different among classes within each factor, Bonferroni analyses were employed in all cases using the GLMM marginal means calculated for each class [37]. GLMM accommodates missing data, so that the 26 chiltepin populations sampled could be included in the analysis. Parallel analyses using only the 8 populations for which data on the 3 years of sampling were available yielded comparable results (data not shown).

The contributions of each ecological factor to the variation in virus and symptom prevalence were estimated using a Principal Component Analysis (PCA). Host plant density (*d*), host plant genetic diversity estimated as expected heterozygosity (*H_e_*), species diversity estimated as species richness (number of species, *SR*) and Shannon index (*Sh*), of 24 chiltepin populations were scaled to zero mean and unit variance, inserted in a regression matrix and rotated to obtain the principal components (PCs). Importantly, species diversity was not measured in TLA-w and HER-cmc, so that these populations were excluded from the analysis. Significance thresholds for the load of each ecological factor on a PC were determined using a broken-stick model [38]. Bivariate analyses, considering both linear and non-linear models, of begomoviruses, CMV and symptom prevalence onto the ecological factors and their corresponding PCs, yielded the proportion of the variance in each of these variables explained by each factor and each principal component (*R^2^*), and the significance of these correlations. For these bivariate analyses, we utilized the GLMM marginal means of begomoviruses, CMV and symptom prevalence calculated for each population. Statistical analyses were performed using the statistical software package SPSS 17.0 (SPSS Inc., Chicago, USA).

Information theory was used to determine the relative importance of the ecological factors in the variation of symptom and virus prevalence [39]. This approach was chosen because it allows making inferences across a set of causal model structures for symptom and virus prevalence [39]. To do so, a set of models that included a global model, which contained all ecological factors (species richness, Shannon index, expected heterozygosity and host plant density), and nested models, which contained different combinations of the predictor variables was fitted. Since species richness and Shannon index always loaded in the same PC, different selection model analyses in which the nested models considered *SR*, or *Sh*, or both variables together were performed. The three approaches gave similar results. For simplicity, only results considering species richness are shown. We ranked the models according to second order Akaike's Information Criteria (AIC_c_) to account for small sample size (R library: AICcmodavg) [39]. The model with the lowest AIC_c_ score was selected as the best-ranked model. We calculated AIC_c_ Delta (Δ_i_), as the difference between the AIC_c_ of a given model and that of the best-ranked model. Delta quantifies how strongly models compete (Δ_i_ = 0 for best-ranked model; Δ_i_ = 1–2 indicates substantial empirical support; Δ_i_ = 4–7 indicates considerable less support; and Δ_i_>10 indicates no support [39]). Finally, the Akaike relative weight (*ω_i_*) of each model was calculated following the expression: *ω_i_* = exp(Δ_i_)/Σexp(Δ_i_).

## Results

### Prevalence of Virus Infection

The status of a total of 1820 censused plants was recorded during the summers of 2007–2009. The prevalence of plants showing symptoms of virus infection (symptomatic plants) (Table 1) marginally varied among year (*χ^2^* = 5.86, *P* = 0.060), ranging between 16.2% and 21.6% of the census.

A subset of 1081 plants, either symptomatic or asymptomatic, was analyzed for infection by ChYMV (a chiltepin-infecting tymovirus, see [36]), CMV, begomoviruses or potyviruses. Low prevalence of potyvirus infection (2.87%) precluded further analyses. ChYMV infection was limited to locations around Tula, AZP (Table 1) where its prevalence was high (42.86%). Infection by CMV and by begomoviruses was detected during the three years of the study in all biogeographical provinces and under different levels of human management (Figure 1 and Table 2). PepGMV and PHYVV were the only begomovirus species detected infecting chiltepin, and their relative prevalence did not depend on the level of human management of the chiltepin population (data not shown). Therefore, from here on these two species will be considered together and referred to as “begomoviruses”. CMV prevalence remained stable (≈7%) among years (*χ^2^* = 0.06, *P* = 0.970), while begomovirus prevalence was about 3–5 times higher (19–36%) and varied largely according to year (*χ^2^* = 58.25, *P*<1×10^−5^) (Table 2).

**Table 2:** Laboratory-determined prevalence of begomoviruses and CMV infection in Mexican chiltepin populations.

		2007			2008			2009		
Habitat^1^	Code^2^	N total^3^	Begomovirus%^4^	CMV%^5^	N total	Begomovirus%	CMV%	N total	Begomovirus%	CMV%
Wild	DZI-W	28	28.6	3.6	27	29.6	7.4			
Wild	HUA-W	35	14.3	0.0	19	31.6	5.3	26	3.9	11.5
Wild	TLA-W	9	11.1	0.0						
Wild	TUL-W				15	26.7	0.0	30	23.3	3.3
Wild	BER-W	48	29.2	8.3	32	34.8	3.1	71	4.2	1.4
Wild	CER-W	5	20.0	0.0				13	15.4	15.4
Wild	HUJ-W							13	46.2	0.0
Wild	PEL-W	43	18.6	7.0	27	11.1	22.2	27	14.8	0.0
Wild	MOC-W							35	2.9	11.4
Wild	MAU-W							31	12.9	0.0
Let standing	TUL-LSF							28	7.1	7.1
Let standing	TUL-LSP				12	58.3	33.3	13	23.1	7.7
Let standing	CER-LSP							20	10.0	20.0
Let standing	ELO-LSP	38	34.2	2.6	34	5.9	0.0	33	3.0	3.0
Let standing	SAN-LSP	24	20.8	16.7				21	57.1	4.8
Let standing	MAZ-LSF							16	0.0	0.0
Cultivated	HUA-CHG	10	20.0	50.0	13	69.3	0.0	22	0.0	9.1
Cultivated	CHO-CHG	18	72.2	5.6	22	50.0	9.1	11	63.6	36.4
Cultivated	TLA-CMC	9	22.2	0.0	16	43.8	6.3	33	3.0	15.2
Cultivated	PVE-CMC	9	77.8	0.0	20	85.0	0.0	20	5.0	0.0
Cultivated	CER-CMC							9	77.8	22.2
Cultivated	POT-CHG							10	60.0	20.0
Cultivated	HUJ-CHG							10	100.0	0.0
Cultivated	LIB-CMC							29	79.3	3.5
Cultivated	TEM-CMC							19	5.3	0.0
Cultivated	HER-CMC							28	3.6	7.1
	**TOTAL**	**276**	**28.6**	**6.9**	**237**	**35.9**	**7.2**	**568**	**18.5**	**6.7**

CMV-infected plants were detected by ELISA-DAS. Begomovirus infected plants were detected by PCR with specific primers in a subset of 1081 plants of the 1820 plants sampled (see Material and Methods).

1 Habitats belonged to three levels of human management.

2 Populations are designated with the first three letters of the name of the nearest village, plus a code indicating the habitat: W = wild, LSP = Let standing, pasture; LSF = Let standing, living fence; CHG = Cultivated, home garden; CMC = Cultivated, monoculture.

3 Total number of analysed plants per population.

4 Percentage of analysed plants detected as begomovirus-infected.

5 Percentage of analysed plants detected as CMV-infected.

Begomoviruses, CMV or ChYMV infection explained the symptoms of 212/281 (78.7%, of these 81% being infected by begomoviruses) laboratory-analyzed symptomatic plants from all populations and years. This fraction did not differ according to the level of human management of the population (59/76 analyzed symptomatic plants for wild populations; 50/70 for let-standing populations, and 103/135 for cultivated populations) (*χ^2^* = 0.86, *P* = 0.651). The fraction of infected plants showing symptoms (212/369, i.e., 57.4% in total) was lower in wild populations (59/133, 44.4%) than in cultivated (103/153, 67.3%) or in let-standing populations (50/83, 60.2%) (*χ^2^*≥4.54, *P*≤0.033). This fraction did not differ between the later two levels of human management (*χ^2^* = 0.89, *P* = 0.345).

### Human Management and Virus Prevalence

The effect of geography (biogeographical province and chiltepin population), and level of human management in the prevalence of symptomatic plants, begomoviruses or CMV, was analyzed. GLMM analyses using biogeographical province as a fixed effect showed that neither the prevalence of symptomatic plants, begomoviruses or CMV did depend on this factor (*F_5,47_*>0.797, *P*<0.557). Similarly, the prevalence of CMV infection did not vary among chiltepin populations (*F_25,47_* = 1.512, *P* = 0.108). However, population was a factor determining the prevalence of symptomatic plants and begomovirus infection (*F_25,47_*>4.369, *P*<1×10^−4^). Bonferroni-corrected multiple comparisons showed that this was solely due to the higher prevalence in populations HUJ-chg and LIB-cmc (*P*<0.046 in 21/25 populations in both cases), and when these populations were removed from the analysis, population was no longer a factor in the prevalence of symptomatic plants and begomovirus infection (*F_23,45_* = 1.984, *P* = 0.183). Populations HUJ-chg and LIB-cmc were not excluded from further analyses in order to consider as much of the variability in the analyzed factors as possible. These results show that the biogeographical factors analyzed largely do not affect viral and symptom prevalence. Consequently, the populations corresponding to each level of human management could be analyzed together.

The level of human management was a factor in determining the prevalence of symptomatic plants, (*F_2,47_* = 7.619, *P*<1×10^−4^), which was significantly lower in wild than in cultivated populations (*P*<1×10^−3^), with let-standing populations showing an intermediate value (*P*≥0.276). Similarly, human management also affected the prevalence of begomovirus infection (*F_2,47_* = 5.774, *P* = 6×10^−4^). Values were higher in cultivated populations than in wild (*P* = 6×10^−4^) and intermediate in let-standing populations (*P*≥0.076). However, CMV prevalence did not vary depending on this factor (*F_2,47_* = 1.459, *P* = 0.243). Thus, increased levels of human management are associated with higher prevalence of symptomatic plants and begomoviruses, but not to prevalence of CMV. We therefore explored which ecological factors varying between populations with different levels of human management were linked to these differences in disease and virus infection risk.

### Ecological Factors and Human Management

The relative importance of focal host plant density (*d*), host genetic diversity (expected heterozygosity, *H_e_*) and species diversity of the habitat expressed either as species richness (*SR*) or considering also species evenness (Shannon index, *Sh*), on chiltepin populations was analyzed by including these variables in a PCA. Relevant statistical parameters of these ecological factors are provided in Table S1 of the Supporting Information. Parallel PCAs were performed considering all populations together and individually for each level of human management (Table 3). Importantly, *SR* and *Sh* loaded in the same PC in all cases. Since both variables represent the same ecological factor, we performed separate PCAs considering either *SR* or *Sh*, but the choice of index did not alter the results (data not shown).

**Table 3:** Principal component analysis of four ecological factors, and their association with symptom, begomovirus and CMV prevalence in chiltepin populations with different levels of human management in Mexico.

Population	All				Wild				Let Standing				Cultivated			
Principal Component	1	2	3	4	1	2	3	4	1	2	3	4	1	2	3	4
***Percentage variance explained in***																
Symptom Prevalence	**18.3** *	9.2	1.3	2.4	**36.8** *	**25.4** *	9.1	0	**71.8** *	7.3	21.3	0.5	**47.1** *	0	8.4	0.9
Begomovirus Prevalence	**16.6** *	0.1	2.2	2.8	6.4	**33** *	1.7	6.7	**35.1** *	0	7.4	13.8	15.2	13.3	4.7	11.3
CMV Prevalence	0	5.6	4.9	4.8	0	0.2	11.8	0	10.9	**40.2** *	**52.7** *	14.6	9.5	3.3	**28.9** *	0.4
***Percent association component-variable***																
Species Richness (*SR*)	**93.6**	0.2	0.0	6.2	**92.4**	3.3	2.3	2.0	1.1	**82.7**	10.6	5.7	8.5	**85.0**	3.8	2.7
Shannon Index (*Sh*)	**81.9**	11.4	4.5	2.2	**84.6**	8.1	3.3	4.0	5.0	**84.7**	5.6	4.7	0.1	**91.1**	3.2	5.7
Plant Density (*d*)	0.4	**86.3**	7.1	6.2	5.8	**89.2**	5.4	0.0	0.2	10.2	**89.6**	0.0	4.9	6.8	**85.5**	2.8
Heterozygosity (*H_e_*)	0.0	5.4	**93.6**	1.0	8.3	13.5	**78.2**	0.0	**99.4**	0.3	0.2	0.1	**87.7**	0.1	7.8	4.5
***Expected values under broken-stick model***	44.0	25.8	26.3	3.9	47.8	28.5	22.2	1.5	44.5	26.5	26.4	2.6	45.7	25.3	25.1	3.9
***Total variance explained by the component***	55.3	27.7	12.7	4.3	73.1	21.8	3.7	1.4	57.9	27.4	12.3	2.4	47.4	35.2	10.3	7.1

Populations: All (n = 24); Wild (n = 9); Let Standing (n = 6); Cultivated (n = 9).

Bold indicates significant association based on broken-stick model thresholds.

*: Significant linear correlation between a PC and prevalence variables (P<0.05).

The PCA using the data set that included all the populations (*All*) yielded three main PCs collectively explaining 95.7 percent of the total variance. Species diversity (*SR* and *Sh*) was highly associated with PC1, *d* with PC2, and *H_e_* with PC3 (Squared loadings>81.9) (Table 3, *All* column). The PCA restricted to wild populations, largely mirrored the results obtained with the *All* data set. However, the fraction of total variance explained by PC1 was higher, and that explained by PC2 and PC3 lower than in the *All* analysis (Table 3, Wild column).

PCAs considering either let-standing or cultivated populations separately yielded PCs explaining similar percentages of the variance than in the *All* data set. However, variables loading in each PC differed from *All* and wild data sets. For let-standing populations, *H_e_* was now associated with PC1, *SR* and *Sh* with PC2, and *d* with PC3 (Squared loadings>82.7) (Table 3, Let-standing column). Similarly, in cultivated populations *H_e_* was associated with PC1, *SR*/*Sh* with PC2, and *d* with PC3 (Squared loadings>85.0) (Table 3, Cultivated column). Importantly, *SR*, *Sh* and *d* loaded positively into their respective PCs, but the loading of *H_e_* was always negative (not shown). The results above indicate that the relative importance of the ecological factors considered in this study vary depending on the level of human management of the chiltepin population.

To determine how human management affects species and genetic diversity, and plant density, we performed GLMM analyses on each PC obtained with the *All* data set using level of human management as a factor. The three major PCs significantly differed depending on the level of human management (*F_2,24_*≥4.995, *P*≤0.015). Values of PC1 (species diversity) were significantly higher in wild than in cultivated populations (*P* = 0.045), with intermediate values in let-standing populations. The opposite trend was observed for PC2 (plant density). For PC3 (host genetic diversity), values for cultivated populations were lower than in let-standing and wild populations (*P*≤0.015), the later two types not differing (*P* = 0.952).

### Association between Ecological Factors and Virus Prevalence

To further explore the association between the considered ecological factors and disease risk, the influence of each ecological factor on symptom, begomovirus and CMV prevalence was studied using model selection analyses. For the *All* data set, symptom, begomovirus and CMV prevalence were chiefly determined by species diversity, either measured as *SR* (Table 4) or *Sh* (not shown). The model including only species richness was unambiguously the best (*ω*>0.79) (Table 4). In wild populations, symptom prevalence was mainly associated with species richness and host density (*ω* = 0.45 and 0.33, respectively), and begomovirus prevalence was chiefly associated with host density (*ω* = 0.40). However the best-ranked model included also host genetic diversity (*ω* = 0.55). Although the best-ranked model explaining CMV prevalence included all the ecological factors, the single factor that best explained the variable was host genetic diversity (*ω* = 0.12) (Table 4). Model selection analyses were largely similar for let-standing and cultivated populations. In both types of populations, host genetic diversity best explained symptom and begomovirus prevalence (*ω* = 0.51 in both cases), with the model including all the ecological factors showing slightly lower weight. Finally, host density chiefly determined CMV prevalence (*ω* = 0.57 and 0.35), but the model considering all factors showed slightly higher weight in cultivated populations (Table 4). Thus, these analyses are in agreement with the PCA.

**Table 4:** Model selection results. For virus and symptoms prevalence, model structures included species diversity (Species richness, *SR*), genetic diversity (expected heterozygosity, *H_e_*), and host plant density (*d*). Best-ranked models are bolded and have the lowest AIC_c_.

Population^1^	All				Wild				Let Standing				Cultivated			
Prevalence & model structure	logLik	AIC_c_ 2	Δ_i_ 3	*ω_i_* 4	logLik	AIC_c_ 2	Δ_i_ 3	*ω_i_* 4	logLik	AIC_c_ 2	Δ_i_ 3	*ω_i_* 4	logLik	AIC_c_ 2	Δ_i_ 3	*ω_i_* 4
***Symptoms Prevalence***																
*SR*	**−0.94**	**5.32**	**0**	**0.852** *	**−2.78**	**3.22**	**0**	**0.447** *	−21.51	33.51	16.37	1.4e−4	−4.17	11.34	3.51	0.058
*H_e_*	−8.54	21.65	16.33	2.4e−4	−7.64	9.70	6.48	0.018	**−14.86**	**17.14**	**0**	**0.507** *	**−2.52**	**7.83**	**0**	**0.334** *
*d*	−9.12	24.35	19.03	6.3e−5	−2.94	3.86	0.64	0.325	−18.1	22.9	5.76	0.028	−5.11	14.02	6.19	0.015
*H_e_+d*	−10.3	27.8	22.48	1.1e−5	−10.7	13.70	10.48	0.002	−16.15	19.85	2.71	0.131	−2.84	8.47	0.64	0.243
*SR+H_e_*	−6.57	17.72	12.40	0.002	−5.34	6.66	3.44	0.080	−20.31	25.31	8.17	0.009	−4.21	11.76	3.93	0.047
*SR+d*	−2.88	15.87	10.55	0.004	−5.53	6.80	3.58	0.075	−19.27	24.27	7.13	0.014	−5.01	13.55	5.72	0.019
*SR+H_e_+d*	2.21	8.91	3.59	0.142	−4.83	6.17	2.95	0.102	−15.14	18.12	0.98	0.311	−2.58	8.16	0.33	0.284
***Begomovirus Prevalence***																
*SR*	**−3.03**	**3.26**	**0**	**0.879** *	−12.35	22.66	13	0.001	−17.08	22.08	15.18	2.6e−4	−6.63	16.26	7.09	0.014
*H_e_*	−11.03	16.63	13.37	0.001	−10.34	15.46	5.8	0.030	**−7.1**	**6.9**	**0**	**0.506** *	**−3.19**	**9.17**	**0**	**0.475** *
*d*	−11.31	17.18	13.92	0.001	−7.53	10.27	0.61	0.402*	−11.33	10.67	3.77	0.077	−5.99	15.98	6.81	0.016
*H_e_+d*	−12.65	18.5	15.24	4.3e−4	**−3.34**	**9.66**	**0**	**0.546**	9.01	10.43	3.53	0.087	−4.34	12.68	3.51	0.082
*SR+H_e_*	−7.60	15.3	12.04	0.002	−12.59	22.75	13.09	0.001	−13.89	14.89	7.99	0.009	−5.43	14.19	5.02	0.039
*SR+d*	−5.95	12.01	8.75	0.011	−9.48	17.85	8.19	0.009	−12.33	13.33	6.43	0.020	−7.22	17.76	8.59	0.006
*SR+H_e_+d*	−7.915	7.5	4.24	0.106	−9.29	17.41	7.75	0.011	−8.06	7.94	1.04	0.301	−3.34	9.68	0.51	0.368
***CMV Prevalence***																
*SR*	**−39.82**	**35.25**	**0**	**0.791** *	−22.05	37.95	12.12	0.001	−21.48	16.38	2.35	0.174	−30.24	32.76	10.55	0.002
*H_e_*	−53.59	44.39	9.14	0.008	−16.88	28.92	3.09	0.116*	−26.12	22.1	8.07	0.010	−33.92	35.92	13.71	4.5e−4
*d*	−42.65	39.32	4.07	0.103	−23.98	39.02	13.19	0.001	**−20.76**	**14.03**	**0**	**0.565** *	−21.27	23.53	1.32	0.351*
*H_e_+d*	−48.92	41.72	6.47	0.031	−15.94	27.06	1.23	0.295	−29.97	26.76	12.73	0.001	−20.41	22.59	0.38	0.220
*SR+H_e_*	−49.65	42.55	7.30	0.021	−25.57	40.76	14.93	3.1e−4	−25.62	21.83	7.80	0.011	−30.52	33.86	11.65	0.001
*SR+d*	−47.6	41.49	6.24	0.035	−22.9	37.43	11.6	0.002	−24.05	20.52	6.49	0.022	−33.16	35.49	13.28	0.001
*SR+H_e_+d*	−51.3	43.73	8.48	0.011	**−14.97**	**25.83**	**0**	**0.546**	−20.17	15.95	1.92	0.216	**−20.59**	**22.21**	**0**	**0.425**

1 All (n = 24); Wild (n = 9); Let Standing (n = 6); Cultivated (n = 9).

2 AIC_c_, second order Akaike's Information Criterion.

3 Where *i* = the model in question and 0 = best ranked model.

4 AIC_c_ model weight; the larger the *ω*, the greater the likelihood of the model given the data, relatively to the competing models [39].

*: Indicates the single ecological factor that best explains the prevalence variables in each type of habitat.

### Effect of Ecological Factors in Virus Prevalence

**Figure 2:**
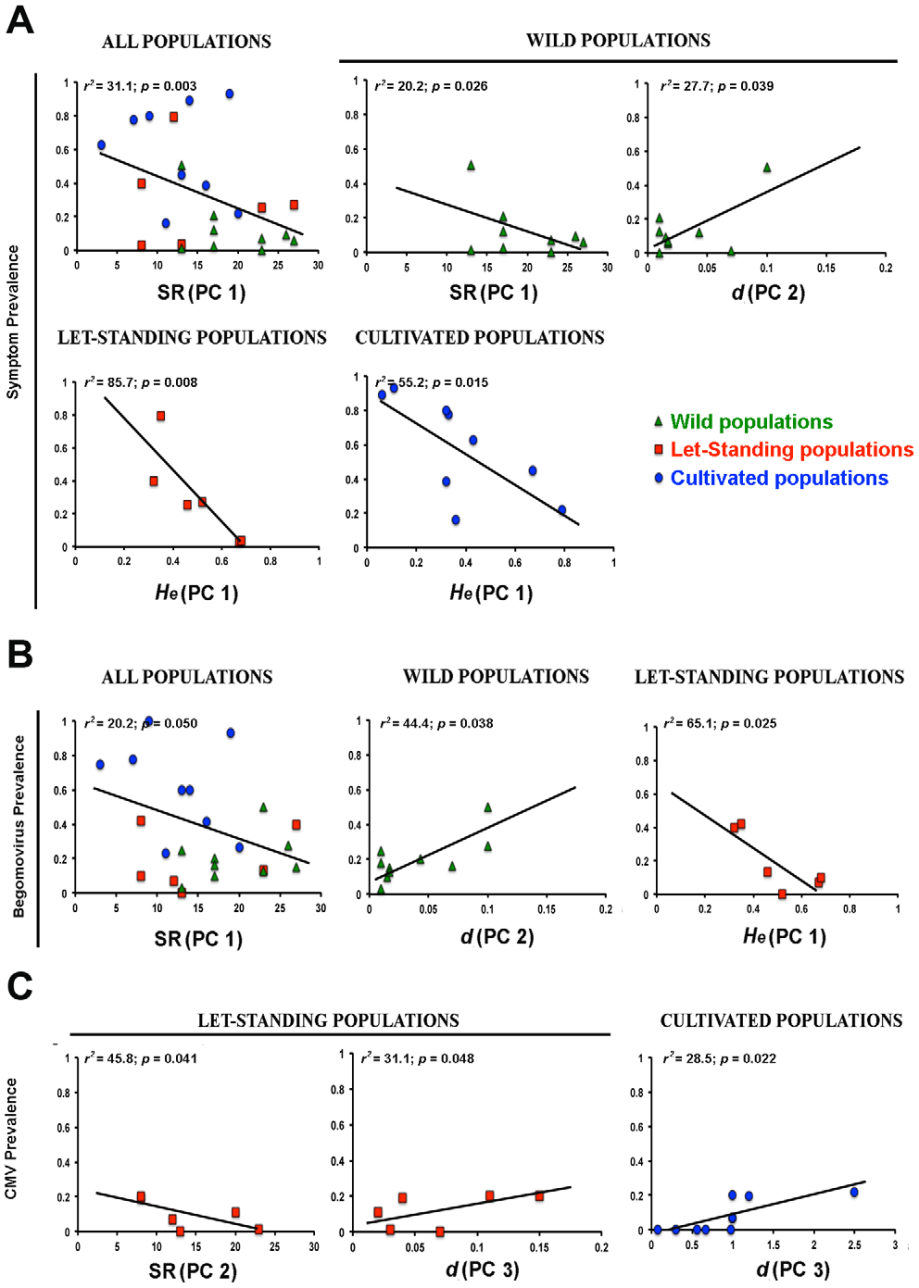
Bivariate relationships between ecological factors and disease/infection risk. Significant regressions of each ecological factor and the prevalence of symptomatic plants (A), and of begomovirus (B) and CMV infection (C), are represented according to the level of human management: Wild (green triangles), let-standing (red squares), and cultivated (blue dots). PCs with the highest association with each ecological factor are shown in parenthesis. *SR* = Species richness expressed as number of species, *H_e_* = Host genetic diversity expressed as expected heterozygosity, *d* = Host plant density. Note the different scales in the X-axis depending on the ecological factor. The Y-axis represents marginal mean prevalence values for each population over the monitored period.

The effect of ecological factors in symptom and virus prevalence was analyzed by bivariate analyses of each factor onto the prevalence of symptomatic plants, begomoviruses and CMV. For the *All* data set, *SR* was negatively associated with symptom and begomovirus prevalence (*P*<0.050), explaining 31.1% and 20.2% of the variance in these variables, respectively (Figure 2 and Table S2). Therefore, species diversity was the primary predictor of symptom and begomovirus prevalence. In wild populations, *SR* explained 20.2% of the variance being negatively associated with symptom prevalence (*P* = 0.026) (Figure 2 and Table S2), and *d* explained 27.7% (*P* = 0.039) of the variance in symptom prevalence, and 44.4% (*P* = 0.038) of the variance in begomovirus prevalence. Thus, in wild populations species diversity had also a principal role in determining symptom prevalence, with a lesser effect of plant density in symptom and begomovirus prevalence. In contrast, in let-standing populations *H_e_* was negatively associated with symptom and begomovirus prevalence (*P*≤0.025), explaining 85.7% and 65.1% of the variance in these two traits, respectively. In addition, a negative correlation between *SR* and CMV prevalence was found (*P* = 0.041, 45.8% of the variance explained), and *d* showed a positive association with CMV prevalence (*P* = 0.048, 31.1% of the variance explained) (Figure 2 and Table S2). Finally, *H_e_* in cultivated populations was also negatively associated with symptom prevalence (*P*≤0.015, 55.2% of the variance explained), and *d* explained 28.5% of the variance in CMV prevalence (*P* = 0.022) (Figure 2 and Table S2). Parallel bivariate analyses using the PCs associated with species richness, Shannon index, expected heterozygosity and host density of each PCA, instead of the original variables yielded similar results (Table 3 and Figure S1).

## Discussion

We have analyzed the prevalence of virus disease and virus infection in populations of a wild plant to test whether increased host density and decreased host genetic diversity in agroecosystems, as compared with wild ecosystems, favors disease risk. These two classical hypotheses of plant pathology [6], [7] are particular cases of a more general one, which is receiving much attention recently, stating that habitat biodiversity is a determinant of disease risk [4] and may be at the root of disease emergence [3], [4]. The wild pepper or chiltepin was the focal host for this study, taking advantage of some unique characteristics of this species. First, wild populations of chiltepin are found in a large variety of habitats in different biogeographical provinces of Mexico [24], [25], which anticipated large differences in species diversity among habitats, as was indeed the case (Table S1). Second, the genetic diversity of wild chiltepin populations differs according to their geographical origin as shown for the 10 wild populations analyzed here [25]. Last, chiltepin populations show different levels of human management, including populations of let-standing plants, which are not sown or planted, but are tolerated or protected in anthropic habitats; and cultivated populations, in which plants are sown in home gardens or in small traditional plots.

The risk of virus disease was estimated as the prevalence of symptomatic plants. Although unapparent virus infection may affect plant fitness [40], we call here diseased plants those showing macroscopic symptoms. This is grounded in our (unpublished) observations of a fecundity reduction in symptomatic plants as compared to both infected or non-infected asymptomatic ones. However, we are aware that prevalence of symptomatic plants may underestimate disease risk, if symptom development were correlated with increased host mortality. Considering these caveats, the risk of disease was positively correlated with the level of human management of the population, being higher in cultivated than in wild populations, and intermediate in let-standing populations. Hence, results support the concept that transition of host habitat from wild ecosystems to agricultural ones results in an increase of disease risk.

A GLMM analysis of the variation of three PCs – associated with species diversity, host genetic diversity and host density – according to the level of human management, strongly suggested that the higher disease risk associated with increased human management is determined by a reduction of biodiversity, both as species diversity and host genetic diversity, and/or by an increased host density. It should be noted that habitat biodiversity and host genetic diversity, estimated as *SR* and *H_e_*, respectively, vary along a continuum over the three levels of human management of chiltepin populations, which should avoid spurious associations. However, we cannot discard that other factors structured according to the level of human management, not specifically addressed in this work, may influence disease risk. Examples could be time of exposure to virus infection, and nutrient availability. Nevertheless, the three PCs associated to the analyzed ecological factors explained more than 95% of the variance of the analyzed variables, regardless that all populations were considered together or differentiating between wild, let-standing and cultivated populations. Therefore, although other ecological factors might have minor effects on disease risk, those here considered accounted for most of the variance within and between levels of human management, and may largely explain the emergence of viral disease associated with human management of chiltepin populations.

More specifically, both PC and model selection analyses showed that, for the *All* and wild data sets, species diversity of the habitat was the major predictor of disease risk (Figure 2, Tables 3 and 4). For let-standing and cultivated populations host genetic diversity was the major predictor of disease risk (Figure 2, Tables 3 and 4). The risk of infection by begomoviruses, which mostly explained symptoms, followed a largely similar pattern, except for a noticeable role of host density in determining virus prevalence in wild populations. These results support the dilution effect hypothesis for a plant-virus system. Moreover, they stress the importance of preserving biodiversity to maintain ecosystem services, a key concept in conservation biology [4]. Results also agree with most analyses of a variety of animal [5], [41] and plant systems [9]–[14], contributing to extend this hypothesis to plant virus diseases. The relationship between biodiversity and disease risk has received comparatively little attention in wild plant-infecting viruses. To our knowledge, Cereal- and Barley yellow dwarf luteoviruses (C/BYDV), which infect many species of grasses and are transmitted in a persistent manner by aphids in a highly species-specific way, is the best characterized system. In this case, most results are compatible with the amplification effect hypothesis, although the complex relationships between grass species, vector multiplication, and virus multiplication/transmission, make the effect of biodiversity on disease risk largely dependent on species composition [18]–[21], [42], [43]. Differences in life histories between luteoviruses and begomoviruses, which cause most symptoms of virus disease in chiltepin (Tables 1 and 2), could explain why effects of biodiversity vary between both systems. Begomoviruses have a narrow host range [29]–[31], and are persistently transmitted by *B. tabaci*, which has a wide host range [32]. Consequently, the larger the species diversity of the habitat, the larger the number of plant species in which *B. tabaci* can feed, and the lower fraction of meals resulting in begomovirus transmission to the focal host, resulting in host encounter reduction (*sensu* [5]). Interestingly, other reports of a dilution effect of biodiversity also refer to persistently transmitted viruses in which the host range of the virus is narrower than that of the vector [44], [45].

Importantly, which of the two different components of biodiversity was the primary predictor of disease and begomovirus infection risk depended on the level of human management. The reduced weight of species diversity in anthropic habitats could be explained by species diversity being largely reduced in cultivated *vs.* wild populations, and not varying largely among let-standing populations (Table S1). Host genetic diversity has been shown to have a negative effect on the risk of fungal diseases in crops [46]–[48]. Results from fungal pathogens were interpreted as due to differences in resistance-susceptibility among host genotypes, resulting in decreased transmission efficiency [46]–[48]. This mechanism could be also invoked to explain our results as differences in resistance to begomovirus infection have been reported among chiltepin genotypes [49]. However, genotype diversity might also reduce pathogen transmission by other mechanisms, for instance, microenvironment changes [13] or modification of the behavior of insect vectors [50].

The reduction of disease and begomovirus-infection risk with higher biodiversity was not coupled to a lower chiltepin density, as host plant density always loaded into a different PC than species or host diversity. An accepted axiom in plant pathology is that higher host density leads to higher disease risk. However, data are scarce and mostly inconclusive [6], [8], [9], and the effects of biodiversity and host density on disease risk are often difficult to differentiate [4]. The few works that attempted to differentiate these effects yielded contrasting results: density was the primary factor determining disease risk [10], [11] or there were independent and complex interactions between the effects of both factors [22]. The methodology used here avoids artificial correlations [51] and allowed disentangling the effects of these two ecological factors on disease and begomovirus infection risk.

Interestingly, infection by CMV followed a different pattern: infection risk did not depend on the level of human management, and host plant density was a relevant parameter in managed populations, but not in wild ones. The different pattern of infection risk found for begomoviruses and CMV could be due to differences in their life histories. At odds with begomoviruses, CMV is a generalist regarding the host and the vector and, perhaps more importantly, it is transmitted in a non-persistent manner. While persistent transmission is effected during feeding periods among plants that are hosts of the aphid vector, non-persistent transmission occurs during probing visits to plants that need not be hosts of the aphids, which remain viruliferous for short periods of time [52]. Thus, proximity of plants susceptible to the virus could be more important than biodiversity in determining CMV infection risk, similarly to directly transmitted fungi infecting leaves [10], [11]. Consequently, the mechanisms of transmission, in addition to the host range of the pathogen and/or its vectors, could be a primary factor in determining the relationship between biodiversity and disease risk, an unexplored issue, to our knowledge.

Finally, a larger fraction of begomovirus- or CMV-infected plants showed symptoms in managed populations than in wild ones, strongly suggesting a higher virulence of virus infection in the former, perhaps due to a higher susceptibility of plants in human-managed populations to virus infection and its effects. The relationship between host physiological condition and disease susceptibility is an underexplored subject [53]. However, we could speculate that plants of managed populations, which benefit from higher levels of water and/or nutrients than those from wild habitats, as shown by their production of about five times as much fruits (our unpublished observations), would be more competent hosts for virus vectors [20], [54]. This would encourage more frequent and longer meals, thus being under higher inoculum pressure of persistently transmitted viruses. Also, a more favorable host condition could result in higher levels of virus multiplication [21], [54], [55]. If this were the case, in addition to suffering more virulent virus infections, plants in cultivated and let-standing populations would be more competent hosts for virus vectors, virus multiplication and transmission. These factors would contribute to the higher disease risk, and thus to disease emergence, in human managed populations, regardless of the ecological factors here analyzed. However, we cannot exclude that the larger proportion of symptomatic plants in managed habitats would be the result of increased life span of infected plants due to the enhanced availability of resources in cultivated and let-standing populations, which could contribute to explain our observations.

In summary, our results show the important role of biodiversity reduction in the emergence of viral diseases associated to human management of plant populations. Our work also suggest that other ecological and genetic factors, perhaps resulting in increased virulence in anthropic habitats, need to be considered in order to fully understand the dynamics of emergence, which should be the subject of future research.

## Supporting Information

Figure S1 **Bivariate relationships between principal components (PCs) associated to ecological factors and disease/infection risk.** Significant regressions of PC and the prevalence of symptomatic plants (A), and of begomovirus (B) and CMV infection (C), are represented according to the level of human management. The X-axis represents the PC as a continuous variable comprised of the principal component scores for each population. Ecological factors with the highest loading on each PC are shown in parenthesis. *SR* = Species richness expressed as number of species, *H_e_* = Host genetic diversity expressed as expected heterozygosity, *d* = Host plant density. The Y-axis represents marginal mean prevalence values for each population over the monitored period.
(TIF)

Table S1 **Relevant statistical parameters of each ecological factor analyzed in Mexican chiltepin populations.**
(DOCX)

Table S2 **Analysis of association between symptoms, begomovirus and CMV prevalence, and ecological factors.**
(DOC)
