# Supplementary figures and images for: Effect of Biodiversity Changes in Disease Risk: Exploring Disease Emergence in a Plant-Virus System
Source: PLoS Pathog. 2012 Jul 5;8(7):e1002796. doi: 10.1371/journal.ppat.1002796 (PMC3390404; doi:10.1371/journal.ppat.1002796)

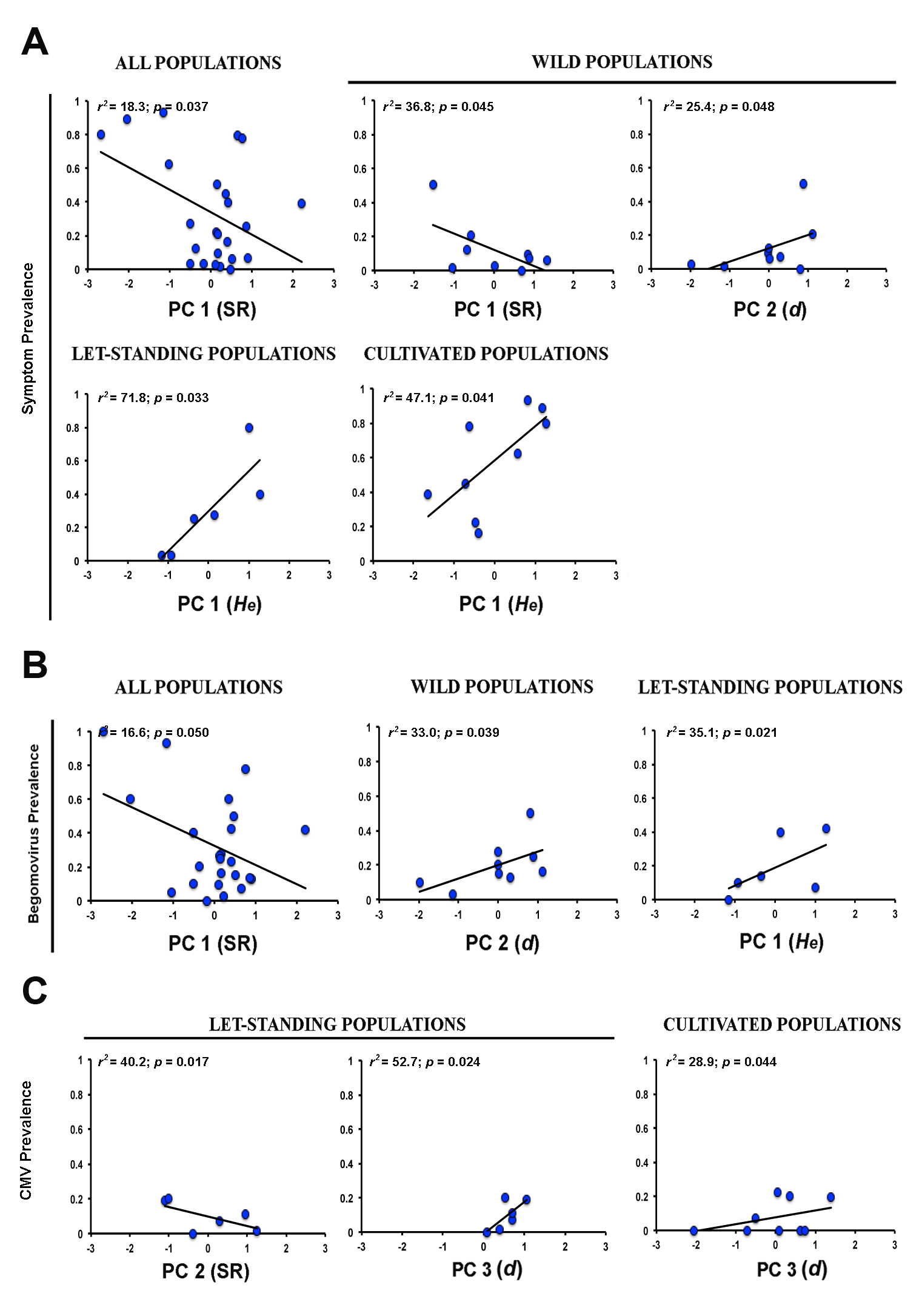

Supplement: Figure S1 — Bivariate relationships between principal components (PCs) associated to ecological factors and disease/infection risk. Significant regressions of PC and the prevalence of symptomatic plants (A), and of begomovirus (B) and CMV infection (C), are represented according to the level of human management. The X-axis represents the PC as a continuous variable comprised of the principal component scores for each population. Ecological factors with the highest loading on each PC are shown in parenthesis. SR = Species richness expressed as number of species, He = Host genetic diversity expressed as expected heterozygosity, d = Host plant density. The Y-axis represents marginal mean prevalence values for each population over the monitored period. (TIF) [file ppat.1002796.s001.tif]
