# Supplementary material for: Effect of Biodiversity Changes in Disease Risk: Exploring Disease Emergence in a Plant-Virus System
Source: PLoS Pathog. 2012 Jul 5;8(7):e1002796. doi: 10.1371/journal.ppat.1002796 (PMC3390404; doi:10.1371/journal.ppat.1002796)
Supplement: Table S1 — Relevant statistical parameters of each ecological factor analyzed in Mexican chiltepin populations. (DOCX) [file ppat.1002796.s002.docx]

**Table S1.** Relevant statistical parameters of each ecological factor analysed in Mexican chiltepin populations.

| **Location^1^** | **Code^2^** | **Region^3^** | **Habitat** | **Ecological Factors** | | | |
| --- | --- | --- | --- | --- | --- | --- | --- |
|  |  |  |  | **Sh^4^** | **SR^5^** | **He^6^** | **d^7^** |
| Dzibilchaltun (YUC) | DZI-W | YUC | wild | 2.78 | 26.00 | 0.75 | 0.02 |
| Cholul (YUC) | CHO-CHG | YUC | home garden | 2.15 | 13.00 | 0.67 | 0.07 |
| Huatulco (OAX) | HUA-W | CPS | wild | 2.97 | 18.86 | 0.66 | 0.02 |
| Huatulco (OAX) | HUA-CHG | CPS | home garden | 2.28 | 20.00 | 0.00 | 0.12 |
| Tlacuapa (SLP) | TLA-W | SMO | wild | ND | ND | 0.47 | 0.01 |
| Tlacuapa (SLP) | TLA-CMC | SMO | monoculture | 1.56 | 11.00 | 0.36 | 1.00 |
| PuertoVerde (SLP) | PVE-CMC | SMO | monoculture | 2.61 | 16.00 | 0.32 | 1.00 |
| Tula (TAM) | TUL-W | AZP | wild | 2.09 | 13.00 | 0.66 | 0.06 |
| Tula (TAM) | TUL-LSF | AZP | live fence | 2.19 | 12.00 | 0.67 | 0.15 |
| Tula (TAM) | TUL-LSP | AZP | pasture | 1.94 | 8.00 | 0.68 | 0.04 |
| Bernal (QRO) | BER-W | AZP | wild | 2.01 | 17.00 | 0.40 | 0.04 |
| Cerritos (SLP) | CER-W | AZP | wild | 2.28 | 17.00 | 0.49 | 0.01 |
| Cerritos (SLP) | CER-LSP | AZP | pasture | 1.45 | 8.00 | 0.35 | 0.11 |
| Cerritos (SLP) | CER-CMC | AZP | monoculture | 1.72 | 7.00 | 0.33 | 0.56 |
| La Libertad (NAY) | LIB-CMC | CPA | monoculture | 0.51 | 2.00 | 0.11 | 2.50 |
| El Potrero (SIN) | POT-CHG | CPA | home garden | 0.60 | 2.00 | 0.06 | 1.00 |
| El Huajote (SIN) | HUJ-W | CPA | wild | 2.72 | 23.00 | 0.51 | 0.01 |
| El Huajote (SIN) | HUJ-CHG | CPA | home garden | 0.53 | 2.00 | 0.70 | 0.30 |
| Puente Elota (SIN) | PEL-W | CPA | wild | 2.91 | 23.00 | 0.57 | 0.02 |
| Elota (SIN) | ELO-LSP | CPA | pasture | 2.87 | 23.00 | 0.46 | 0.03 |
| Sanalona (SIN) | SAN-LSP | CPA | pasture | 2.50 | 27.00 | 0.52 | 0.02 |
| Moctezuma (SON) | MOC-W | SON | wild | 2.05 | 13.00 | 0.41 | 0.07 |
| Mazocaui (SON) | MAZ-LSF | SON | live fence | 1.86 | 13.00 | 0.32 | 0.07 |
| Los Mautos (SON) | MAU-W | SON | wild | 2.15 | 17.00 | 0.16 | 0.10 |
| Temporal (SON) | TEM-CMC | SON | monoculture | 0.70 | 3.00 | 0.43 | 0.67 |
| Hermosillo (SON) | HER-CMC | SON | monoculture | ND | ND | 0.32 | 1.00 |
|  |  |  |  |  |  |  |  |
|  |  | **Pooled** | **Wild (W)** | **2.44 ± 0.04** | **19.56 ± 0.53** | **0.61 ± 0.02** | **0.04 ± 0.01** |
|  |  | **Pooled** | **Let-Standing (LS)** | **2.22 ± 0.09** | **16.29 ± 1.31** | **0.50 ± 0.03** | **0.06 ± 0.02** |
|  |  | **Pooled** | **Cultivated (C)** | **1.41 ± 0.08** | **8.44 ± 0.68** | **0.34 ± 0.03** | **0.82 ± 0.07** |
|  |  |  | **TOTAL** | **2.01 ± 0.03** | **14.64 ± 0.31** | **0.44 ± 0.01** | **0.33 ± 0.02** |

^1^Population names. Abbreviations of the department in which the population is located are shown in parenthesis.

^2^Populations are named with the three first letters of the name of the nearest village, plus a code indicating the habitat: W= wild, LSP= Let standing, pasture; LSF= Let standing, living fence; CHG= Cultivated, home garden; CMC= Cultivated, monoculture.

^3^YUC: Yucatán; CPS: Costa del Pacífico Sur; SMO: Sierra Madre Oriental; AZP: Altiplano Zacatecano-Potosino; CPA: Costa del Pacífico; SON: Sonora.

^4^Mean Shannon Index.

^5^Mean Species Richness.

^6^Mean observed heterozygosity.

^7^Mean plant density.
