# Supplementary material for: Effect of Biodiversity Changes in Disease Risk: Exploring Disease Emergence in a Plant-Virus System
Source: PLoS Pathog. 2012 Jul 5;8(7):e1002796. doi: 10.1371/journal.ppat.1002796 (PMC3390404; doi:10.1371/journal.ppat.1002796)
Supplement: Table S2 — Analysis of association between symptoms, begomovirus and CMV prevalence, and ecological factors. (DOC) [file ppat.1002796.s003.doc]

Table S2. Analysis of association between symptoms, begomovirus and CMV prevalence, and ecological factors.

| Population | **All** | |  | **Wild** | |  | **Let-Standing** | |  | **Cultivated** | |
| --- | --- | --- | --- | --- | --- | --- | --- | --- | --- | --- | --- |
|  | ***r*** | ***P*** |  | ***r*** | ***P*** |  | ***r*** | ***P*** |  | ***r*** | ***P*** |
| ***Symptom Prevalence*** |  |  |  |  |  |  |  |  |  |  |  |
| Species Richness (*SR*) | **-55.7*** | **0.003** |  | **-44.7*** | **0.026** |  | -2.2 | 0.939 |  | -17.3 | 0.642 |
| Shannon Index (*Sh*) | **-66.3*** | **0.000** |  | -36.1 | 0.148 |  | -31.6 | 0.549 |  | -3.2 | 0.955 |
| Heterozygosity (*H_e_*) | 1.1 | 0.789 |  | -36.2 | 0.300 |  | **-92.7*** | **0.008** |  | **-74.2*** | **0.015** |
| Plant Density (*d*) | 38.7 | 0.262 |  | **52.6*** | **0.039** |  | 40.1 | 0.428 |  | 35.9 | 0.305 |
|  |  |  |  |  |  |  |  |  |  |  |  |
| ***Begomovirus Prevalence*** |  |  |  |  |  |  |  |  |  |  |  |
| Species Richness (*SR*) | **-44.5*** | **0.021** |  | -35.8 | 0.334 |  | -26.5 | 0.625 |  | -16.9 | 0.678 |
| Shannon Index (*Sh*) | **-46.9*** | **0.021** |  | -31.6 | 0.400 |  | -22.4 | 0.675 |  | -22.0 | 0.538 |
| Heterozygosity (*H_e_*) | 4.5 | 0.954 |  | -35.2 | 0.304 |  | **-80.6*** | **0.025** |  | -11.0 | 0.353 |
| Plant Density (*d*) | 9.5 | 0.673 |  | **66.1*** | **0.038** |  | 1.0 | 0.870 |  | 19.9 | 0.593 |
|  |  |  |  |  |  |  |  |  |  |  |  |
| ***CMV Prevalence*** |  |  |  |  |  |  |  |  |  |  |  |
| Species Richness (*SR*) | -22.1 | 0.306 |  | 7.1 | 0.983 |  | **-67.8*** | **0.041** |  | -14.8 | 0.737 |
| Shannon Index (*Sh*) | 6.3 | 0.791 |  | -14.1 | 0.755 |  | **-55.7*** | **0.048** |  | -33.2 | 0.393 |
| Heterozygosity (*H_e_*) | -17.7 | 0.421 |  | -22.8 | 0.546 |  | -28.3 | 0.595 |  | -31.1 | 0.369 |
| Plant Density (*d*) | 9.9 | 0.566 |  | 1.3 | 0.961 |  | **62.4*** | **0.018** |  | **53.9*** | **0.022** |

* Significant linear correlation between prevalence variables and ecological factors (*P*<0.05).
